# Increased Phenotypic Plasticity to Climate May Have Boosted the Invasion Success of Polyploid *Centaurea stoebe*

**DOI:** 10.1371/journal.pone.0050284

**Published:** 2012-11-20

**Authors:** Min A. Hahn, Mark van Kleunen, Heinz Müller-Schärer

**Affiliations:** 1 Department of Biology, Ecology and Evolution, University of Fribourg, Fribourg, Switzerland; 2 Department of Biology, Ecology, University of Konstanz, Konstanz, Germany; Federal University of Rio de Janeiro, Brazil

## Abstract

Phenotypic plasticity may allow organisms to cope with altered environmental conditions as e.g. after the introduction into a new range. In particular polyploid organisms, containing more than two sets of chromosomes, may show high levels of plasticity, which could in turn increase their environmental tolerance and invasiveness. Here, we studied the role of phenotypic plasticity in the invasion of *Centaurea stoebe* (Asteraceae), which in the native range in Europe occurs as diploids and tetraploids, whereas in the introduced range in North America so far only tetraploids have been found. In a common garden experiment at two sites in the native range, we grew half-sibs of the three geo-cytotypes (native European diploids, European tetraploids and invasive North American tetraploids) from a representative sample of 27 populations. We measured the level and the adaptive significance of phenotypic plasticity in eco-physiological and life-history traits in response to the contrasting climatic conditions at the two study sites as well as three different soil conditions in pots, simulating the most crucial abiotic differences between the native and introduced range. European tetraploids showed increased levels of phenotypic plasticity as compared to diploids in response to the different climatic conditions in traits associated with rapid growth and fast phenological development. Moreover, we found evidence for adaptive plasticity in these traits, which suggests that increased plasticity may have contributed to the invasion success of tetraploid *C. stoebe* by providing an advantage under the novel climatic conditions. However, in invasive tetraploids phenotypic plasticity was similar to that of native tetraploids, indicating no evolution of increased plasticity during invasions. Our findings provide the first empirical support for increased phenotypic plasticity associated with polyploids, which may contribute to their success as invasive species in novel environments.

## Introduction

A crucial pre-requisite of an invader is its ability to survive and reproduce in the new environment. Therefore, it has often been suggested that phenotypic plasticity, the ability of a genotype to express different phenotypes depending on environmental conditions, and the potentially associated increased environmental tolerance [1], [2], [3], may contribute to the invasiveness of a species [4], [5]. In particular for sessile plants this may be important, as they cannot escape from unfavorable conditions. Moreover, if more plastic genotypes have a fitness advantage over less plastic genotypes, and thus, phenotypic plasticity is adaptive [6], [7], it might not only provide an advantage in the initial phases of colonization and establishment, but it may further increase in the introduced range through selection [5], [8].

Indeed, several studies suggested phenotypic plasticity as a potential mechanism for plant invasions [9]. However, two recent meta-analyses comparing plasticity between invasive and non-invasive or native species came to contradicting conclusions about its general importance for invasions [10], [11]. These variable outcomes of studies that compared species may partly reflect the complexity of phenotypic plasticity, which is a property of a single genotype with regard to a specific trait under a specific set of environmental conditions. Therefore, in-depth studies on potential causes of intra-specific variation in plasticity and its ecological consequences are needed, and may increase the mechanistic understanding of the role of plasticity in invasions.

Polyploidy, the state of an organism containing more than two sets of chromosomes per cell, has often been suggested to increase phenotypic plasticity [12]. It is a widespread phenomenon [13], and can have several direct and indirect effects on the genetics, morphology, physiology and ecology of an organism [14], [15], [16]. Through genetic and genomic rearrangements, polyploidy may also affect the genetic mechanisms underlying phenotypic plasticity, such as overdominance, pleiotropy or epistasis [3], [17], [18]. Moreover, besides selection and drift, it has been suggested that a main process underlying the evolution of plasticity may involve the disruption of the genetic system through hybridization or polyploidization [2]. As a potential consequence, polyploids may show an increased range of phenotypic responses resulting in their often reported large ecological tolerance [14]. This may allow them to occupy wider geographic ranges compared to their diploid conspecifics [19], [20] and eventually also increase their invasiveness under novel environmental conditions. Indeed, several studies suggest polyploidy as a beneficial attribute of invasive species [21], [22], [23], [24], [25]. In addition to various other processes that may underlie the invasion success of polyploids [see 25], phenotypic plasticity could play an important role, but so far it has remained largely unexplored. In particular, empirical tests of the effects of polyploidy on the level of phenotypic plasticity, on its potential adaptive significance and how it may affect the invasiveness of a species under the novel environmental conditions, may provide important insights into potential mechanisms underlying the success of polyploids.

In this study, we explored the role of phenotypic plasticity and polyploidy in the invasion of *Centaurea stoebe* L. (spotted knapweed). Although several other factors may contribute to the invasion success of this species [see e.g. 26,27], we focused here on phenotypic plasticity, which so far has remained a largely unexplored aspect in the invasion of *C. stoebe*. In the native range in Europe, *C. stoebe* occurs as two cytotypes, diploids (EU 2x) and tetraploids (EU 4x), while in the introduced range in North America so far only tetraploids (NA 4x) have been found [24], [28]. In the following, we refer to these three cytotype × continent combinations as geo-cytotypes. In addition to this disparate cytotype distribution between the two ranges, a large shift in the climatic niche in North American tetraploids towards warmer and more continental conditions has been reported [29]. Therefore we compared phenotypic plasticity in response to these particular conditions between diploids and tetraploids from Europe to explore whether polyploidy might increase plasticity, and thus pre-adapt tetraploids to become invasive [26]. Furthermore, we compared plasticity between native European and invasive North American tetraploids, which may indicate potential rapid evolutionary changes or, alternatively, genetic drift due to founder effects [26] affecting the level of phenotypic plasticity during the invasion process.

We performed a common garden experiment at two sites in the native range simulating the climatic differences between the two ranges in combination with different soil conditions (three resource levels in pots). This allowed us to broadly disentangle above-ground (e.g. temperature and light) and below-ground (soil water content and nutrient availability) effects. To determine the level of plasticity in key eco-physiological traits related to water and resource use, as well as life-history traits (including fitness-related traits as e.g. biomass or number of flowers), we grew half-sibs of the three geo-cytotypes under the different experimental conditions. Specifically, we asked (1) whether the measured traits respond plastically to the different climate and soil conditions, (2) whether phenotypic plasticity is associated with fitness benefits, and (3) whether the three geo-cytotypes differ in phenotypic plasticity in these traits.

## Materials and Methods

### Study Species

*Centaurea stoebe* L. (syn. *C. maculosa* Lam., Asteraceae) is a perennial herb that grows in a wide range of habitats, typically in dry grassland and disturbed or slightly open vegetation [30]. In its native range in Europe, it occurs as two cytotypes, diploids (2n = 18) and tetraploids (2n = 36), mostly in single-cytotype populations [28]. Recent molecular analyses revealed an allopolyploid origin of the tetraploids (polyploidization involving interspecific hybridization), with genome contributions of diploid *C. stoebe* as well as a second currently unknown parental species [31]. The two cytotypes differ in morphology [28] and life-cycle. Diploids are mostly biennial and monocarpic, whereas tetraploids are perennial, polycarpic and already start flowering in the first year [26], [28], [32], [33].

In the late 19^th^ century, *C. stoebe* was accidentally introduced to North America as seed contaminant of alfalfa seeds, where it has become a highly aggressive rangeland weed, causing enormous ecological and economic damage [34]. Although most likely both cytotypes have been introduced to North America due to their largely overlapping geographical range in Europe and the clear evidence for multiple introduction [35], only tetraploids have been found there so far [24].

### Common Garden Experiment

We used seeds of *C. stoebe* from our seed stock, which includes a large number of natural populations across the native (diploid and tetraploid) and introduced range (tetraploids only) that had been collected during a survey in 2005 [for details see 24]. No specific permissions were required, as the locations were not privately-owned or protected in any way and there was no involvement of endangered or protected species. To assure robust comparisons among the three geo-cytotypes, we carefully selected a representative sample (Table S1) consisting of three populations from each of three different geographical regions per geo-cytotype with high similarities in their environmental conditions (based on ecological niche models; [24]). A similar sampling approach was recently used for a demography study [27]. This sampling design was based on multivariate analyses including 93 native (diploid and tetraploid) and 48 invasive populations (tetraploids only) and thus, representing various conditions of the major part of the distributional range [see also 27]. Moreover, despite of the sample covering only three geographical regions from each continent, they include in particular the core regions of the invasion of *C. stoebe* in the introduced range, i.e. the US Pacific North-West [30]. To capture the variation among and within populations, we used six individuals (half-sibs) from each of three mother plants from each of the nine populations per geo-cytotype, resulting in a total of 486 plants (3 geo-cytotypes×3 regions×3 populations×3 seed families×6 individuals).

Single seeds were planted into 2×2 cm cells in 150-cell seedling trays filled with nutrient-poor horticultural soil (TKS1, Floragard, Germany) in April 2009, and kept regularly watered in an uncontrolled glasshouse at the University of Fribourg, Switzerland. After four weeks, the seedlings were re-potted into 2-L pots with three different soil conditions (low water/low nutrient, high water/low nutrient, high water/high nutrient; see Table S2 for more information on soil properties in the different treatments). These soil treatments were chosen to create a gradient with different resource levels that may occur within and between the native and introduced ranges [24]. For the low water treatment, we used a 1∶1 mixture of horticultural soil and sand. In the high water treatment, 15% water-retaining crystals (Dyofix Water Crystals, Industrial Dyes and Dyesstuff, UK) were added to the equivalent amount of the soil mixture to increase soil moisture, with negligible expected effects on soil properties other than moisture. Effective volumetric soil water content (Table S2) in the pots was measured several times after periods of different length without precipitation using a soil humidity probe (HH2, Delta-T Devices, Cambridge, UK). In the high nutrient treatment, we added 100 ml of diluted (10 mg N/L) liquid fertilizer (Wuxal, Maag, Switzerland) every second week during the vegetation period (Table S2). Equal amounts of water were added to the pots from the other treatments.

By the end of May 2009, the plants were transferred into two experimental gardens. As determined by climatic niche models [29], [36], the site in Fribourg (N 46° 47.506’, E 7° 9.519’, 643 m a.s.l.) represents the generally more moderate climate of the native range, while the site in Conthey (N 46° 12.504’, E 7° 18.137’, 479 m a.s.l.) shows a high climatic match with the continental climate in most of the introduced range in North America (see e.g. summer temperature and precipitation; Figure S1). Mean annual precipitation and temperature in 2009 and 2010 was 865 mm and 8.7°C for the site in Fribourg and 555 mm and 10.6°C for the site in Conthey (data from AGROMETEO, Swiss federal administration).

At both sites, the plants were arranged in a randomized block design with three blocks. The six individuals of each seed family were assigned to different blocks as well as different soil treatments at each site. Each block contained 81 plants representing all geo-cytotypes, regions, populations and seed families. Pots were spaced by a distance of 0.5 m and buried to ground level to prevent heating by the sun. Despite of the insertion of a special cloth at the bottom of each pot, some roots grew out of the pots. After three weeks, we added a second layer of cloth and a second pot leaving a gap between pot and ground. Initially, all pots within a site received equal small amounts of water to assure survival.

### Measurements

On a subset of 81 plants per site, representing all individuals of three seed families from one randomly chosen population per geo-cytotype and region, we measured a set of eco-physiological traits related to water and resource use. Stomatal conductance, i.e. the rate of gas exchange through the stomata (mainly uptake of CO_2_ for photosynthesis and transpiration of water vapour; [37]), was measured on two intact and fully developed leaves per plant using a porometer (AP4, Delta-T Devices, Cambridge, UK). In 2009, measurements were taken on 1^st^ September in Fribourg and 11^th^ September in Conthey. In 2010, measurements were taken on 5^th^ July in Fribourg and 22^nd^ June in Conthey. In both years, the same individuals were measured and all measurements were taken under similar, moderate and stable weather conditions. Furthermore, we measured leaf traits on two fully developed, intact leaves of similar phenological stage per plant. Leaves were collected on 13^th^ to 15^th^ July 2009 and 6^th^ July 2010 in Fribourg, and on 20^th^ July 2009 and 23^rd^ June 2010 in Conthey and immediately enclosed in plastic bags with some droplets of water for 24 hours. Fully turgescent leaves were weighed (fresh weight), scanned for leaf area (analyzed with MideBMP software, Version 4.2) and dried for 48 hours at 60°C to determine dry weight. Leaf dry matter content (LDMC) was calculated as the ratio of leaf dry weight to fresh weight and specific leaf area (SLA) by dividing leaf area by leaf dry weight [38], [39]. In 2010, combined samples of two leaves per plant were grinded and analyzed using Mass Spectrometry in the Isolab at the Institute of Plant Sciences ETH, Zurich, Switzerland. We determined carbon and nitrogen contents as well as water-use-efficiency by carbon isotope discrimination Δ [40].

Furthermore, we measured several life-history traits on all plants on 19^th^ and 26^th^ October 2009 and 9^th^ and 10^th^ August 2010 in Fribourg, and on 28^th^ to 29^th^ October 2009 and 2^nd^ to 4^th^ August 2010 in Conthey. We recorded survival, number of accessory rosettes (secondary rosettes giving rise to new shoots in the following season) and shoots as well as the height of the largest shoot. Total number of buds, flowerheads (open capitula) and seedheads (withered flowerheads containing seeds) per plant were counted (in 2009) or estimated (in 2010) by multiplying the number of shoots by the average number of buds, flowerheads and seedheads of the smallest, an intermediate and the largest shoot. For each year, a phenology index (0 to 1; 0: early stage, only buds present; 1: late stage, all flowers wilted and mature seeds produced) was calculated using the following formula: 0.5 * (*f*/(*b*+*f*+*s*))+(*s*/(*b*+*f*+*s*)) with the number of buds *b*, the number of flowerheads *f* and the number of seedheads *s*. Fertile flowers were counted on three flowerheads per plants collected from the terminal ends of the second (one flowerhead) and third branch (two flowerheads) on 14^th^ October 2009 and 9^th^ and 10^th^ August 2010 in Fribourg, and in 7^th^ October 2009 and 3^rd^ August 2010 in Conthey. Fecundity per plant was estimated by multiplication of total number of capitula by the average number of fertile flowers per flowerhead. Aboveground biomass (excluding accessory rosettes bearing new shoots for the upcoming season) was harvested on 12^th^ November 2009 and 7^th^ and 8^th^ September 2010 in Fribourg, and on 13^th^ November 2009 and 21^st^ to 23^rd^ August 2010 in Conthey. In 2009, the entire harvested biomass was dried for at least 48 hours at 60°C and weighed. As the plants were large in 2010, we measured the fresh weight of the entire biomass directly in the field and dried approximately 30–50% of the biomass of each plant to determine the individual water content, which we used to derive the total dry weight of each individual plant.

### Statistical Analyses

We first explored general differences in traits among geo-cytotypes and treatments as well as differences in absolute phenotypic plasticity among geo-cytotypes (indicated by significant geo-cytotype×treatment interactions) including all data points to get robust comparisons using linear and generalized linear mixed effects models (LMM and GLMM). We performed two separate sets of analyses for the comparisons of EU 2x *vs*. EU 4x and EU 4x *vs*. NA 4x. Models were fitted using geo-cytotype, site, soil treatment (water, nutrients) and all their interactions as fixed and seed family nested in population nested in region as well as block as random factors (population was excluded from the random part for variables measured only on the subset in which there was only one population per region). Model selection was done by removing non-significant terms based on likelihood ratio tests (LRT) with restricted maximum likelihood (REML) estimations for random effects and maximum likelihood (ML) estimations for fixed effects. Parameter estimates were calculated using REML. Models were fitted with the ‘lmer’ function in the ‘lme4’ package in R [41] using the identity link function for normally distributed data and the logit link function for binomial data.

The adaptive significance of plasticity was tested using three different approximations for fitness: biomass, number of capitula and number of flowers (both cumulative values as well as values of the year in which the trait was measured). To explore general adaptive significance of plasticity for each trait and treatment (site, water, nutrient), we fitted linear models (LM) on the combined data of all three geo-cytotypes using for each family the standardized (mean = 0, SD = 1) mean fitness across environments as response variable and the standardized values of the absolute plasticity (mean of environment 1 minus mean of environment 2), the standardized mean trait values and geo-cytotype as explanatory variables. The standardized mean trait values were included to disentangle the fitness effect of the average value (i.e. the elevation of the reaction norm) from the fitness effect of plasticity (i.e. slope of the reaction norm) of a trait. A positive estimate (slope) for plasticity indicates adaptive plasticity (increasing fitness with increasing relative plasticity), whereas a negative estimate indicates maladaptive plasticity [42].

To explicitly compare phenotypic plasticity among geo-cytotypes, we calculated a relative plasticity index for each seed family, trait and each specific treatment (site, water, nutrients). We restricted our analyses to complete data pairs from individuals of the same seed family in the contrasting environments that are tested. For each pair, we subtracted the trait value in the environment with the lower mean trait values across all seed families from the trait value in the environment with the higher mean trait values across all seed families, and divided this by the mean of the two values to obtain a relative plasticity index. For each seed family, we calculated an average relative plasticity index across all pairs of the same seed family. To test for differences in relative plasticity indices among geo-cytotypes, separate analyses were performed for each trait, comparison (EU 2x *vs*. EU 4x and EU 4x *vs*. NA 4x) and treatment (site, water, nutrient). We fitted LMMs using geo-cytotype as fixed and population nested in region as random factors (population was excluded for traits measured only on the subset). Selection of the random part was performed with LRTs on REML estimations. Parameter estimates were calculated using REML. Models were fitted using the ‘lme’ function in the ‘nlme’ package in R [43]. All analyses were performed with the statistical software R version 2.9.2 [44].

## Results

### Trait Differences Among Geo-cytotypes and Treatments

European diploids and European tetraploids showed differences in traits mainly reflecting the different life-cycles (Table S3). In particular, on average tetraploids had a lower LDMC (EU 2x: 211±5 mg/g; EU 4x: 199±4 mg/g (mean ± se), *P*<0.003) and carbon content (EU 2x: 46.7±0.2%; EU 4x: 46.2±0.2% (mean ± se), *P*<0.02) compared to diploids. Furthermore, the number of capitula per year (EU 2x: 524±116; EU 4x: 885±108 (mean ± se), *P*<0.001), total number of capitula (EU 2x: 527±103; EU 4x: 946±73 (mean ± se), *P*<0.001) and total biomass (EU 2x: 129±45 g; EU 4x: 191±28 g (mean ± se), *P*<0.009) were higher in tetraploids.

North American tetraploids on average showed higher carbon isotope discrimination compared to European tetraploids (EU 4x: 22.6±0.2; NA 4x: 23.1±0.2 (mean ± se), *P*<0.005), indicating lower water use efficiency. Moreover, a larger number of plants in North American tetraploids produced accessory rosettes in the second year (EU 4x: 9.3±5.4%; NA 4x: 27.4±6.6% (mean ± se), *P*<0.001) and North American plants showed accelerated phenology compared to European tetraploids (EU 4x: 0.71±0.10; NA 4x: 0.91±0.03 (mean ± se), *P*<0.002).

In general, plant responses to the different site conditions were pronounced (Table S3), with consistently higher performance in Conthey. In addition to climatic differences among the sites, the average soil water content differed significantly between the two sites (CO: 15.9±0.2% (mean ± se); FR: 21.9±0.2% (mean ± se); LR = 237.39, *P*<0.0001). However, we did not detect any significant difference in soil water content between the two water treatments within each site (*P*>0.05) and only a few traits showed differences between the soil treatments (water, nutrients) (Table S3).

### Adaptive Significance of Plasticity

Our results revealed adaptive phenotypic plasticity in several traits, however, depending on the measure of fitness (Table S4). Plastic responses to the different site treatments were more consistently adaptive (23 trait × fitness combinations adaptive, 5 maladaptive, 101 in total) as compared to responses to the soil conditions (water treatment: 15 trait × fitness combinations adaptive, 13 maladaptive, 101 in total; nutrient treatment: 8 trait × fitness combinations adaptive, 13 maladaptive, 96 in total). In particular, plasticity in SLA, LDMC and phenology was adaptive (Table S4).

### Differences in Plasticity Among geo-cytotypes

**Figure 1:**
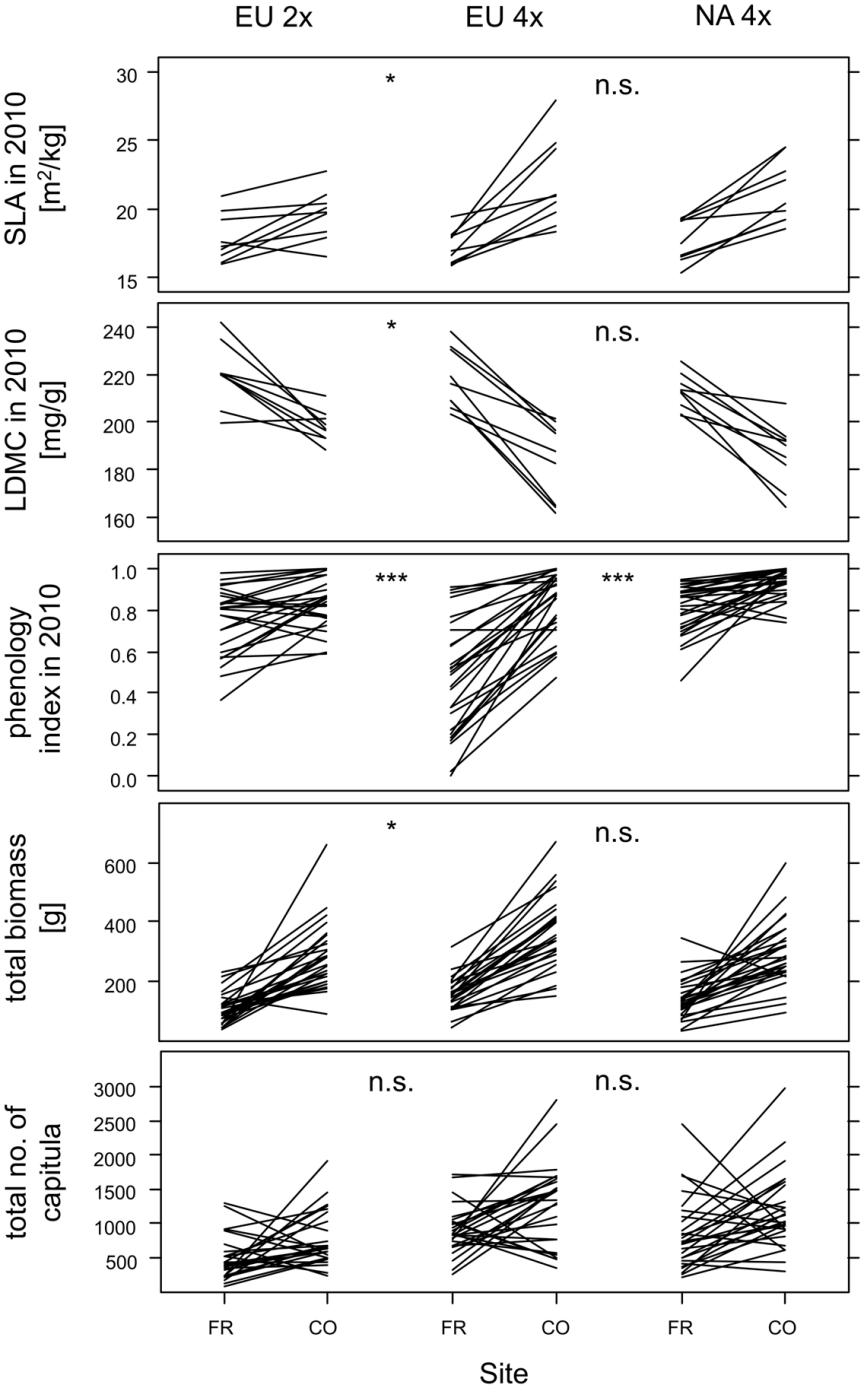
Reaction norms of selected traits of the three geo-cytotypes of *C. stoebe* in response to the different site conditions. Each line represents the mean response of a single seed family to different site conditions (FR: Fribourg; CO: Conthey). Differences in absolute plasticity between geo-cytotypes are given by: n.s.: *P*≥0.05, *: *P*<0.05, **: *P*<0.01, ***: *P*<0.001 (from mixed effects models).

We found evidence for differences in phenotypic plasticity in response to different climatic conditions between European diploids and tetraploids in several traits (Table S3 and Table S5). Absolute and relative plasticity was higher in European tetraploids compared to European diploids in specific leaf area (in 2010), leaf dry matter content (in 2010) and phenology in both years (Table S3 and Table S5, Fig. 1). Moreover, relative plasticity in stomatal conductance (*P*<0.04), as well as absolute plasticity in the number of shoots (in 2010: *P*<0.001) and cumulative biomass (*P*<0.05) was higher in European tetraploids than in diploids. In contrast, compared to European tetraploids, diploids showed larger relative plasticity in shoot height in 2009 (*P*<0.01) and larger absolute plasticity in biomass in 2009 (*P*<0.03) in response to the site treatment. However, these two latter results were based on only few replicates of diploids (4 seed families), as most diploids did not bolt in 2009 (Table S3 and Table S5).

We also found differences in plasticity among geo-cytotypes in response to the different soil conditions in a few traits (Table S3 and Table S5). In particular, absolute and relative plasticity of phenology in 2009 in response to the different water treatments differed between diploids and tetraploids (*P*<0.04; Table S3 and Table S5). However, the graphical exploration of the reaction norms revealed no consistent patterns across seed families in none of these traits.

Compared to European tetraploids, North American tetraploids showed lower absolute and relative plasticity in response to the site treatment in phenology in 2010 (Fig. 1, Table S3 and Table S5). Moreover, absolute plasticity in the number of shoots (*P*<0.03) and biomass in 2009 (*P*<0.02) was lower in North American than in European tetraploids (Table S3).

In response to the soil treatments, we found differences in absolute and relative plasticity between European and North American tetraploids in a few traits (Table S3 and Table S5). But again, the graphical exploration of the reaction norms revealed no consistent patterns across seed families in these traits.

## Discussion

We found evidence for increased phenotypic plasticity in polyploid *C. stoebe* in traits related to rapid growth and fast phenological development in response to climatic conditions. In addition to the generally increased performance and fitness of tetraploids (e.g. increased life-time fecundity associated with polycarpy) as well as other potential drivers of the invasion success (e.g. altered biotic interactions such as reduced levels of competition and herbivory), increased plasticity in response to the specific climatic conditions may have contributed to the success of the invasion of tetraploids in North America. However, although our results suggest plasticity in these traits to be adaptive, no further increase in plasticity in North American tetraploids was observed.

We are well aware that some of the traits in this study may (by definition) be intercorrelated and therefore the results must be interpreted with caution. Nevertheless, we report these individual traits, as each of them may have slightly different ecological significance. Moreover, as field-collected seeds were used in this study, we cannot exclude that differences among geo-cytotypes, populations or genotypes are due to maternal effects. But according to previous studies on maternal effects in *C. stoebe*, they are expected to be low as compared to genetic and environmental effects [45].

### Increased Phenotypic Plasticity in Tetraploid C. stoebe

Increased phenotypic plasticity in polyploids has often been suggested as a potential mechanism underlying their generally broader ecological tolerance as compared to diploids [19]. However, empirical studies that explicitly compared levels of plasticity among different cytotypes of a species are rare [46], and do not provide general support for this hypothesis [e.g. 47,48,49]. The increased plasticity in tetraploid as compared to diploid *C. stoebe* in this study suggests that polyploidy may increase and maintain higher levels of plasticity in this species. This is well in line with expectations from the previously reported larger realized ecological niche in tetraploid as compared to diploid *C. stoebe* [24], and may act as a potential mechanism, which could at least partly contribute to the different ecological amplitudes of the two cytotypes.

Aside from genetic and genomic rearrangements following polyploidization [15], [16], in particular the acquisition of new genes through the hybrid origin of allo-tetraploid *C. stoebe* [31], may have affected the level of phenotypic plasticity [2]. Moreover, in the specific case of *C. stoebe* also different evolutionary histories as well as the different life-cycles associated with the two cytotypes [26] could affect the degree of phenotypic plasticity. Due to the longer generation time, plasticity might generally be more important for tetraploids than for short-living diploids, which may adapt more rapidly to changing environments [50], [51].

### Phenotypic Plasticity May Pre-adapt Tetraploids for Invasion

In line with several previous studies that suggested a link of phenotypic plasticity to invasiveness of a species [see e.g. 5], our findings suggest that high levels of plasticity in European tetraploid *C. stoebe* in response to the specific climatic conditions of the introduced range may have pre-adapted them to become invasive. In addition to general differences in traits between the two cytotypes, which may provide tetraploids an advantage under the warmer and more continental conditions in the introduced range in North America [26], [29], we found increased plasticity in tetraploids as compared to diploids in traits associated with rapid growth and fast phenological development. In particular the plastic responses of specific leaf area and leaf dry matter content indicate the ability of tetraploids to rapidly produce biomass under the simulated conditions of the introduced range in North America. A similar pattern was also found in the phenology of these plants either through direct selection or alternatively, through correlation among traits. Rapid growth associated with high specific leaf area and earlier phenological development are generally expected to favor colonization and invasion [e.g. 52,53,54], especially under the specific climatic conditions in most of the introduced range in North America with dry and hot summers, which may further accelerate the phenology of plants [55]. In line with this, our analyses revealed that these responses are adaptive and therefore suggest that plasticity in these traits may indeed have contributed to the success of tetraploids under the novel conditions in the introduced range. Moreover, increased plasticity in these traits may allow tetraploids to tolerate more continental conditions also in their native range in Europe [24], and contribute to their more recent spread in Europe [56], [57].

Although increased phenotypic plasticity in tetraploid *C. stoebe* may have contributed to its invasion success, we cannot conclude from our study that the difference in plasticity between tetraploids and diploids determines the absence of the latter in North America. In particular, we expected phenotypic plasticity to increase fitness homeostasis through elevated environmental tolerance (e.g. increased tolerance of water stress associated with high temperatures). But the generally higher plant performance of all geo-cytotypes in Conthey as compared to Fribourg despite of the drier soil rather resembles a pattern of opportunism [in the sense of 5]. This suggests that plants in Conthey might not have been limited by water, but in turn may have particularly benefited from higher temperatures, which is commonly found in plants [58], [59].

Despite of the prevalence of plastic responses in *C. stoebe* in response to climatic differences, we cannot fully disentangle above-ground (climate) *vs*. belowground (soil) effects as these may be correlated to a certain degree (e.g. precipitation, temperature and soil moisture). The more consistent and generally larger effects of the site as compared to the soil treatment in this study are partially in line with findings of a recent meta-analysis [10], in which differences in plasticity between native and invasive species to different nutrient conditions were generally smaller than differences in plasticity to different water or light conditions. In our study, however, we found no consistent differences in soil humidity by adding the water-retaining crystals. This could theoretically result from unequal water uptake across different treatments. However, as we generally found only few effects of the soil treatments on plant traits, we believe that the similarity in soil humidity among treatments might either be an artifact of the discrete measurements in time after different lengths of periods without rain, or indicate that the treatment was not as effective as in a previous pilot-experiment under controlled greenhouse conditions. In addition, root escape from the pots may have interfered with soil treatments, therefore the general absence of plasticity in response to the soil treatments cannot be interpreted as no effect. Nevertheless, our climate-matching approach using multivariate modeling to determine the experimental sites [24], [29] allowed us to explore phenotypic plasticity in response to multiple and presumably the most relevant environmental factors, rather than along an over-simplistic single factor gradient [60], and thus, provides robustness and realism to our results.

### No Further Increase in Plasticity in Introduced Tetraploids

It has been suggested that adaptive plasticity conferring a fitness advantage may be beneficial for invasions and in turn is likely to further evolve in the introduced range [5]. A recent study on *Polygonum caespitosum* indeed reported evidence for high plasticity in invasive populations, which may have been favored rather than the evolution of locally specialized populations [61]. Also, a study comparing native and introduced plants of *Plantago lanceolata* in five common gardens along an altitudinal gradient showed that the introduced ones had evolved a higher climatic tolerance [62], suggesting that they have increased plasticity of physiological and/or morphological traits. Contrary to our expectations and despite of the evidence for adaptive significance in some traits under the simulated conditions of the introduced range (in particular in specific leaf area, leaf dry matter content and phenology), we found no evidence for further increased plasticity in invasive North American tetraploids. However, North American tetraploids also showed generally earlier development, and therefore may already be well adapted to the conditions in the introduced range in North America. Nevertheless, to test if evolutionary changes may have occurred, ongoing molecular marker studies are needed to determine the source populations of North American tetraploids in Europe, which will allow more robust comparisons. In addition, our measure of the adaptive significance of plasticity strongly depended on the (approximated) fitness, and therefore overall evidence for both adaptive and maladaptive significance of plasticity in this study remains ambiguous and further studies are necessary. Nevertheless, phenotypic plasticity may have acted as a bridge to conquer the novel environment. Moreover, as recently evidenced in a multi-year common garden experiment comparing the demography of the three geo-cytotypes, earlier phenology and more rapid growth may increase population growth rates [27], which reflect the most comprehensive measures of fitness and ultimately determines the success of biological invasions [63].

### Conclusion

This is the first study that explicitly links polyploidy and invasiveness of a species to phenotypic plasticity as a potential mechanism [25] and to our knowledge the first empirical evidence for increased phenotypic plasticity in polyploids. Although also other factors may play an important role for the success of tetraploids in North America, our findings indicate that polyploidy and its associated changes in the genetics, physiology and life-history could increase and maintain higher levels of phenotypic plasticity in ecologically meaningful traits, which may have contributed to the successful establishment of tetraploid *C. stoebe* under the specific set of conditions in the introduced range in North America.

## Supporting Information

Figure S1 **Experimentally simulated climatic conditions of the native** ***vs*** **. introduced range of** ***C. stoebe*** **.** A) Monthly average temperatures and B) precipitation in the main growing season (April to September). Fribourg represents the climatic conditions of the native range in Europe (lower summer temperature, higher precipitation), Conthey simulates the climatic conditions of the introduced range in North America (higher summer temperature, lower precipitation) and Missoula shows the climatic conditions in the core area of the introduced range in North America (higher summer temperature, lower precipitation).
(TIF)

Table S1 **Origin of seed material.** Source populations of maternal plants of *C. stoebe* used in the experiment from each of three different eco-geographical regions in the native and introduced range.
(DOC)

Table S2 **Properties of soils used in the experiment.** (a) nutrient contents in the three soil treatments; initial: nutrient content per pot (2 L) (equal amounts of TKS1 in all treatments); added: total nutrients per pot sequentially added over two growing seasons) and total: cumulative nutrient amounts per pot. (b) average soil humidity in the tree soil treatments at the two experimental sites.
(DOC)

Table S3 **Traits comparisons among geo-cytotypes and experimental treatments.** Results of mixed effects model analyses (LMM, GLMM) after model selection for all traits for the two separate analyses with data of (a) European diploids and European tetraploids and (b) European tetraploids and North American tetraploids. Significant terms (*P*<0.05) based on likelihood ratio tests are shown in bold. Geo-cytotype×site or geo-cytotype×soil interactions indicate differences in absolute phenotypic plasticities between the geo-cytotypes.
(DOC)

Table S4 **Adaptive significance of phenotypic plasticity.** Summary of tests on adaptive significance of phenotypic plasticity of *C. stoebe* in traits in response to site conditions (significant results highlighted in bold) based on different fitness measures (biomass, number of capitula and flowers; cumulative and for individual years).
(DOC)

Table S5 **Comparisons of relative phenotypic plasticity among geo-cytotypes.** Differences in relative phenotypic plasticity indices (PI) between (a) European diploid and European tetraploid and (b) European tetraploid and North American tetraploid *C. stoebe* in response to different experimental treatments (site, water, nutrients).
(DOC)
