# Supplementary figures and images for: Increased Phenotypic Plasticity to Climate May Have Boosted the Invasion Success of Polyploid Centaurea stoebe
Source: PLoS One. 2012 Nov 20;7(11):e50284. doi: 10.1371/journal.pone.0050284 (PMC3502303; doi:10.1371/journal.pone.0050284)

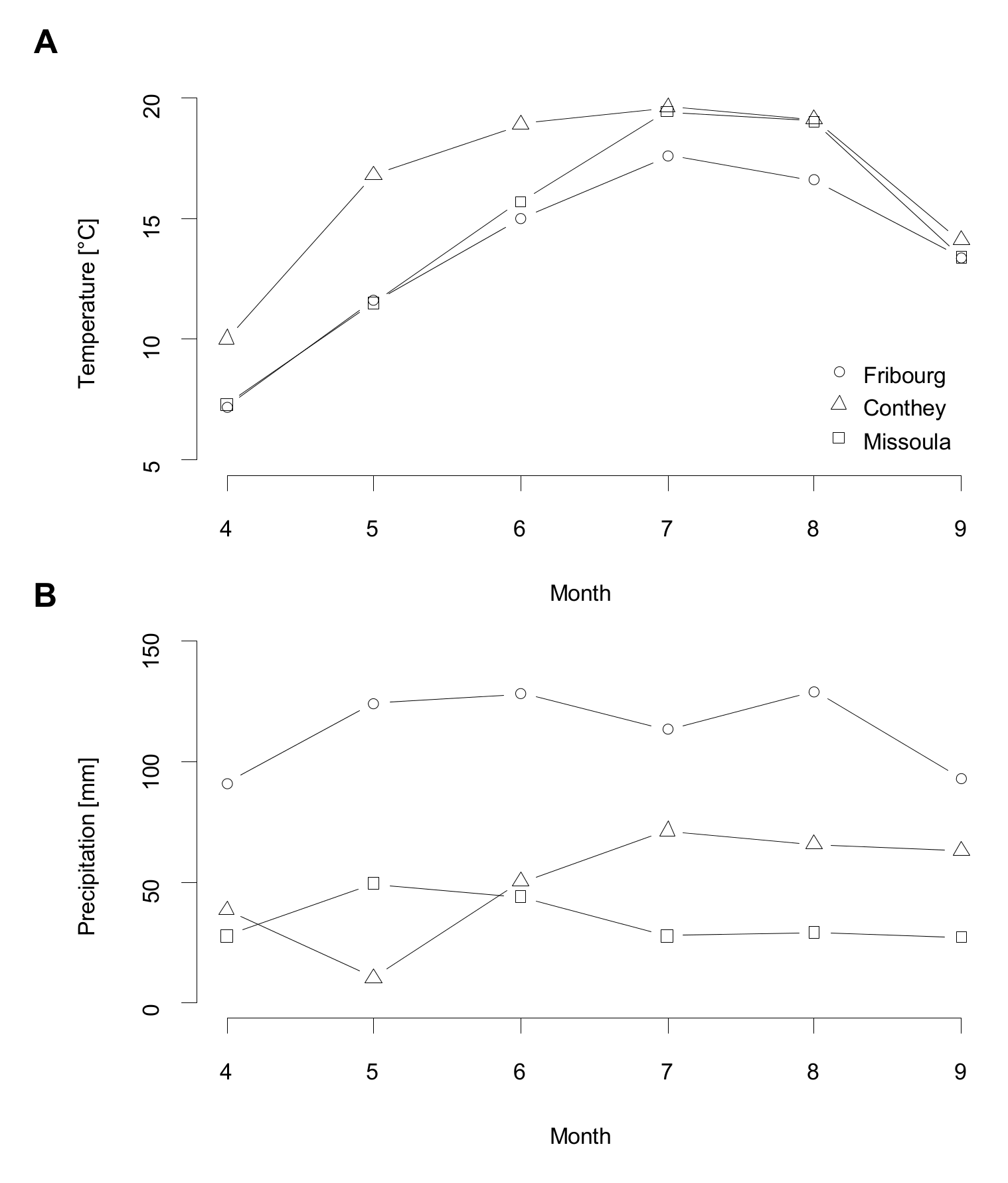

Supplement: Figure S1 — Experimentally simulated climatic conditions of the native vs . introduced range of C. stoebe . A) Monthly average temperatures and B) precipitation in the main growing season (April to September). Fribourg represents the climatic conditions of the native range in Europe (lower summer temperature, higher precipitation), Conthey simulates the climatic conditions of the introduced range in North America (higher summer temperature, lower precipitation) and Missoula shows the climatic conditions in the core area of the introduced range in North America (higher summer temperature, lower precipitation). (TIF) [file pone.0050284.s001.tif]
