# Supplementary material for: Increased Phenotypic Plasticity to Climate May Have Boosted the Invasion Success of Polyploid Centaurea stoebe
Source: PLoS One. 2012 Nov 20;7(11):e50284. doi: 10.1371/journal.pone.0050284 (PMC3502303; doi:10.1371/journal.pone.0050284)
Supplement: Table S1 — Origin of seed material. Source populations of maternal plants of C. stoebe used in the experiment from each of three different eco-geographical regions in the native and introduced range. (DOC) [file pone.0050284.s002.doc]

**Supporting Table S1**

| **Pop Code** | **Region** | **Continent** | **Ploidy** | **Latitude** | **Longitude** |
| --- | --- | --- | --- | --- | --- |
| SUAC | EU-1 | EU | 2x | N 49° 13.223’ | E 24° 42.294’ |
| SUAH | EU-1 | EU | 2x | N 49° 40.239’ | E 33° 42.038’ |
| SUAI | EU-1 | EU | 2x | N 49° 40.051’ | E 34° 56.887’ |
| SUAA | EU-1 | EU | 4x | N 48° 8.281’ | E 23° 4.604’ |
| SUAD | EU-1 | EU | 4x | N 48° 15.012’ | E 25° 53.787’ |
| UA4 | EU-1 | EU | 4x | N 48° 30.955‘ | E 26° 27.948’ |
| D1 | EU-2 | EU | 2x | N 48° 15.639’ | E 13° 0.903’ |
| DE2 | EU-2 | EU | 2x | N 47° 39.714’ | E 7° 31.821’ |
| DE11 | EU-2 | EU | 2x | N 49° 10.283’ | E 11° 57.953’ |
| DE3 | EU-2 | EU | 4x | N 49° 25.010’ | E 11° 5.139’ |
| DE4 | EU-2 | EU | 4x | N 49° 59.620’ | E 10° 37.886’ |
| DE5 | EU-2 | EU | 4x | N 50° 17.894’ | E 10° 39.454’ |
| H1 | EU-3 | EU | 2x | N 46° 43.321’ | E 17° 46.133’ |
| H3 | EU-3 | EU | 2x | N 46° 54.846’ | E 17° 20.098’ |
| H5 | EU-3 | EU | 2x | N 46° 6.536’ | E 18° 55.511’ |
| H2 | EU-3 | EU | 4x | N 47° 6.994’ | E 17° 26.604’ |
| H4 | EU-3 | EU | 4x | N 45° 57.913’ | E 17° 29.998’ |
| SHE | EU-3 | EU | 4x | N 46° 24.095’ | E 17° 28.405’ |
| USMT5 | NA-1 | NA | 4x | N 44° 51.319’ | W 111° 23.663’ |
| USMT6 | NA-1 | NA | 4x | N 45° 17.758’ | W 110° 49.917’ |
| USMT9 | NA-1 | NA | 4x | N 47° 18.034’ | W 112° 7.540’ |
| USMT11 | NA-2 | NA | 4x | N 47° 18.502’ | W 114° 17.985’ |
| USOR7 | NA-2 | NA | 4x | N 44° 33.821’ | W 121° 25.096’ |
| USOR8 | NA-2 | NA | 4x | N 44° 3.305’ | W 121° 14.644’ |
| USOR2 | NA-3 | NA | 4x | N 45° 41.883’ | W 121° 30.340’ |
| USOR10 | NA-3 | NA | 4x | N 42° 14.260’ | W 121° 47.740’ |
| USOR11 | NA-3 | NA | 4x | N 44° 9.400’ | W 122° 15.735’ |
