# Supplementary material for: Increased Phenotypic Plasticity to Climate May Have Boosted the Invasion Success of Polyploid Centaurea stoebe
Source: PLoS One. 2012 Nov 20;7(11):e50284. doi: 10.1371/journal.pone.0050284 (PMC3502303; doi:10.1371/journal.pone.0050284)
Supplement: Table S2 — Properties of soils used in the experiment. (a) nutrient contents in the three soil treatments; initial: nutrient content per pot (2 L) (equal amounts of TKS1 in all treatments); added: total nutrients per pot sequentially added over two growing seasons) and total: cumulative nutrient amounts per pot. (b) average soil humidity in the tree soil treatments at the two experimental sites. (DOC) [file pone.0050284.s003.doc]

**Supporting Table S2**

| **(a) soil nutrients** |  |  |  |  |  |  |  |  |  |
| --- | --- | --- | --- | --- | --- | --- | --- | --- | --- |
|  | **initial** |  |  | **added** |  |  | **total** |  |  |
| **soil treatment** | **N [mg]** | **P_2_O_5_ [mg]** | **K_2_O [mg]** | **N [mg]** | **P_2_O_5_ [mg]** | **K_2_O [mg]** | **N [mg]** | **P_2_O_5_ [mg]** | **K_2_O [mg]** |
| low water / low nutrient | 140 | 80 | 190 | 0 | 0 | 0 | 140 | 80 | 190 |
| high water / low nutrient | 140 | 80 | 190 | 0 | 0 | 0 | 140 | 80 | 190 |
| high water / high nutrient | 140 | 80 | 190 | 120 | 120 | 90 | 260 | 200 | 280 |
| **(b) soil humidity** |  |  |  |  |  |  |  |  |  |
|  | **site** |  |  |  |  |  |  |  |  |
|  | **Fribourg** |  |  | **Conthey** |  |  |  |  |  |
| **soil treatment** | **mean [%]** | **±** | **s.e. [%]** | **mean [%]** | **±** | **s.e. [%]** |  |  |  |
| low water / low nutrient | 22.04 | ± | 0.51 | 15.49 | ± | 0.32 |  |  |  |
| high water / low nutrient | 21.89 | ± | 0.44 | 16.40 | ± | 0.35 |  |  |  |
| high water / high nutrient | 21.77 | ± | 0.47 | 15.90 | ± | 0.39 |  |  |  |
