# Supplementary material for: Increased Phenotypic Plasticity to Climate May Have Boosted the Invasion Success of Polyploid Centaurea stoebe
Source: PLoS One. 2012 Nov 20;7(11):e50284. doi: 10.1371/journal.pone.0050284 (PMC3502303; doi:10.1371/journal.pone.0050284)
Supplement: Table S3 — Traits comparisons among geo-cytotypes and experimental treatments. Results of mixed effects model analyses (LMM, GLMM) after model selection for all traits for the two separate analyses with data of (a) European diploids and European tetraploids and (b) European tetraploids and North American tetraploids. Significant terms (P<0.05) based on likelihood ratio tests are shown in bold. Geo-cytotype×site or geo-cytotype×soil interactions indicate differences in absolute phenotypic plasticities between the geo-cytotypes. (DOC) [file pone.0050284.s004.doc]

**Supporting Table S3**

| **(a) EU 2x *vs*. EU 4x** | |  |  |  |  |  |  |  |  |  |  |  |  |  |  |  |  |  |  |  |
| --- | --- | --- | --- | --- | --- | --- | --- | --- | --- | --- | --- | --- | --- | --- | --- | --- | --- | --- | --- | --- |
|  | |  | **model terms** | | | | | | | | | | | | | | | | | |
|  | |  | **geo-cytotype** | | | **site** | | | **soil** | | | **geo-cytotype × site** | | | **geo-cytotype × soil** | | | **site × soil** | | |
| **trait** | **analysis** | **trans.** | **df** | **Chisq** | ***P*** | **df** | **Chisq** | ***P*** | **df** | **Chisq** | ***P*** | **df** | **Chisq** | ***P*** | **df** | **Chisq** | ***P*** | **df** | **Chisq** | ***P*** |
| C | LMM | - | 1 | 5.74 | **0.017** | 1 | 19.72 | **0.000** | - | - | - | - | - | **-** | - | - | **-** | - | - | **-** |
| N | LMM | - | - | - | - | 1 | 7.68 | **0.006** | 2 | 7.21 | **0.027** | - | - | - | - | - | - | - | - | - |
| SLA1 | LMM | - | - | - | - | 1 | 37.99 | **0.000** | - | - | - | - | - | - | - | - | - | - | - | - |
| SLA2 | LMM | - | 1 | 1.93 | 0.165 | 1 | 4.98 | **0.026** | - | - | - | 1 | 5.00 | **0.025** | - | - | - | - | - | - |
| LDMC1 | LMM | - | - | - | - | 1 | 28.32 | **0.000** | - | - | - | - | - | - | - | - | - | - | - | - |
| LDMC2 | LMM | - | 1 | 9.05 | **0.003** | 1 | 35.43 | **0.000** | - | - | - | 1 | 4.89 | **0.027** | - | - | - | - | - | - |
| SC1 | LMM | log | - | - | - | 1 | 10.68 | **0.001** | - | - | - | - | - | - | - | - | - | - | - | - |
| SC2 | LMM | log | - | - | - | 1 | 8.96 | **0.003** | - | - | - | - | - | - | - | - | - | - | - | - |
| ΔC | LMM | - | - | - | - | - | - | - | - | - | - | - | - | - | - | - | - | - | - | - |
| surv1 | GLMM | - | - | - | - | 1 | 9.35 | **0.002** | 2 | 11.73 | **0.003** | - | - | - | - | - | - | - | - | - |
| surv2 | GLMM | - | - | - | - | 1 | 0.92 | 0.337 | 2 | 2.26 | 0.322 | - | - | - | - | - | - | 2 | 7.68 | **0.022** |
| presros1 | GLMM | - | - | - | - | - | - | - | - | - | - | - | - | - | - | - | - | - | - | - |
| presros2 | GLMM | - | 1 | 12.19 | **0.000** | - | - | - | - | - | - | - | - | - | - | - | - | - | - | - |
| ros1 | LMM | sqrt | 1 | 2.74 | 0.098 | 1 | 81.12 | **0.000** | 2 | 0.16 | 0.921 | - | - | - | 2 | 6.54 | **0.038** | - | - | - |
| ros2 | - | - | - | - | - | - | - | - | - | - | - | - | - | - | - | - | - | - | - | - |
| bolt1 | GLMM | - | 1 | 8.56 | **0.003** | - | - | - | - | - | - | - | - | - | - | - | - | - | - | - |
| bolt2 | GLMM | - | - | - | - | 1 | 4.81 | **0.028** | 2 | 6.02 | **0.049** | - | - | - | - | - | - | - | - | - |
| shoots1 | GLMM | - | - | - | - | 1 | 19.01 | **0.000** | - | - | - | - | - | - | - | - | - | - | - | - |
| shoots2 | GLMM | - | 1 | 25.35 | **0.000** | 1 | 7.91 | **0.005** | 2 | 2.97 | 0.227 | 1 | 14.46 | **0.000** | 2 | 12.73 | **0.002** | - | - | - |
| height1 | LMM | - | - | - | - | - | - | - | 2 | 8.83 | **0.012** | - | - | - | - | - | - | - | - | - |
| height2 | LMM | - | - | - | - | 1 | 12.45 | **0.000** | - | - | - | - | - | - | - | - | - | - | - | - |
| phen1 | GLMM | - | 1 | 0.09 | 0.767 | 1 | 2.53 | 0.112 | 2 | 7.75 | **0.021** | 1 | 181.88 | **0.000** | 2 | 45.41 | **0.000** | 2 | 26.60 | **0.000** |
| phen2 | GLMM | - | 1 | 3.46 | 0.063 | 1 | 13.33 | **0.000** | 2 | 18.99 | **0.000** | 1 | 502.00 | **0.000** | 2 | 68.27 | **0.000** | 2 | 53.74 | **0.000** |
| biomass1 | LMM | log | 1 | 0.01 | 0.923 | 1 | 2.02 | 0.156 | - | - | - | 1 | 5.24 | **0.022** | - | - | - | - | - | - |
| biomass2 | LMM | log | - | - | - | 1 | 8.98 | **0.003** | - | - | - | - | - | - | - | - | - | - | - | - |
| totbiomass | LMM | log | 1 | 7.03 | **0.008** | 1 | 6.80 | **0.009** | - | - | - | 1 | 3.94 | **0.047** | - | - | - | - | - | - |
| capitula1 | LMM | sqrt | - | - | - | - | - | - | - | - | - | - | - | - | - | - | - | - | - | - |
| capitula2 | LMM | sqrt | 1 | 11.11 | **0.001** | - | - | - | - | - | - | - | - | - | - | - | - | - | - | - |
| totcapitula | LMM | sqrt | 1 | 42.32 | **0.000** | - | - | - | - | - | - | - | - | - | - | - | - | - | - | - |
| flowperhead1 | LMM | - | 1 | 19.71 | **0.000** | - | - | - | 2 | 5.39 | 0.068 | - | - | - | 2 | 10.97 | **0.004** | - | - | - |
| flowperhead2 | LMM | - | 1 | 20.69 | **0.000** | - | - | - | - | - | - | - | - | - | - | - | - | - | - | - |
| flowers1 | LMM | log | - | - | - | - | - | - | - | - | - | - | - | - | - | - | - | - | - | - |
| flowers2 | LMM | log | - | - | - | - | - | - | - | - | - | - | - | - | - | - | - | - | - | - |
| totflowers | LMM | log | - | - | - | - | - | - | - | - | - | - | - | - | - | - | - | - | - | - |
| **(b) EU 4x *vs*. NA 4x** | |  |  |  |  |  |  |  |  |  |  |  |  |  |  |  |  |  |  |  |
|  | |  | **model terms** | | | | | | | | | | | | | | | | | |
|  | |  | **geo-cytotype** | | | **site** | | | **soil** | | | **geo-cytotype × site** | | | **geo-cytotype × soil** | | | **site × soil** | | |
| **trait** | **analysis** | **trans.** | **df** | **Chisq** | ***P*** | **df** | **Chisq** | ***P*** | **df** | **Chisq** | ***P*** | **df** | **Chisq** | ***P*** | **df** | **Chisq** | ***P*** | **df** | **Chisq** | ***P*** |
| C | LMM | - | - | - | - | 1 | 5.02 | **0.025** | - | - | - | - | - | - | - | - | - | - | - | - |
| N | LMM | - | - | - | - | 1 | 7.03 | **0.008** | - | - | - | - | - | - | - | - | - | - | - | - |
| SLA1 | LMM | - | 1 | 1.53 | 0.216 | 1 | 7.90 | **0.005** | 2 | 0.37 | 0.832 | - | - | - | 2 | 10.47 | **0.005** | - | - | - |
| SLA2 | LMM | - | - | - | - | 1 | 45.48 | **0.000** | - | - | - | - | - | - | - | - | - | - | - | - |
| LDMC1 | LMM | - | - | - | - | 1 | 8.58 | **0.003** | - | - | - | - | - | - | - | - | - | - | - | - |
| LDMC2 | LMM | - | - | - | - | 1 | 89.37 | **0.000** | - | - | - | - | - | - | - | - | - | - | - | - |
| SC1 | LMM | log | - | - | - | 1 | 9.73 | **0.002** | - | - | - | - | - | - | - | - | - | - | - | - |
| SC2 | LMM | log | - | - | - | 1 | 8.04 | **0.005** | - | - | - | - | - | - | - | - | - | - | - | - |
| ΔC | LMM | - | 1 | 8.10 | **0.004** | - | - | - | - | - | - | - | - | - | - | - | - | - | - | - |
| surv1 | GLMM | - | - | - | - | 1 | 5.02 | **0.025** | - | - | - | - | - | - | - | - | - | - | - | - |
| surv2 | GLMM | - | - | - | - | - | - | - | - | - | - | - | - | - | - | - | - | - | - | - |
| presros1 | GLMM | - | - | - | - | - | - | - | - | - | - | - | - | - | - | - | - | - | - | - |
| presros2 | GLMM | - | 1 | 18.93 | **0.000** | 1 | 2.72 | 0.099 | 2 | 3.36 | 0.186 | - | - | - | 2 | 6.25 | **0.044** | - | - | - |
| ros1 | LMM | sqrt | - | - | - | 1 | 71.68 | **0.000** | - | - | - | - | - | - | - | - | - | - | - | - |
| ros2 | LMM | sqrt | - | - | - | 1 | 10.77 | **0.001** | - | - | - | - | - | - | - | - | - | - | - | - |
| bolt1 | GLMM | - | - | - | - | - | - | - | - | - | - | - | - | - | - | - | - | - | - | - |
| bolt2 | GLMM | - | - | - | - | 1 | 4.82 | **0.028** | 2 | 6.02 | **0.049** | - | - | - | - | - | - | - | - | - |
| shoots1 | GLMM | - | 1 | 3.68 | 0.055 | 1 | 18.18 | **0.000** | - | - | - | 1 | 5.30 | **0.021** | - | - | - | - | - | - |
| shoots2 | GLMM | - | 1 | 3.91 | **0.048** | 1 | 8.56 | **0.003** | 2 | 16.17 | **0.000** | - | - | - | - | - | - | - | - | - |
| height1 | LMM | - | 1 | 0.59 | 0.442 | - | - | - | 2 | 0.66 | 0.717 | - | - | - | 2 | 8.38 | **0.015** | - | - | - |
| height2 | LMM | - | - | - | - | 1 | 11.67 | **0.001** | - | - | - | - | - | - | - | - | - | - | - | - |
| phen1 | GLMM | - | 1 | 1.95 | 0.163 | 1 | 7.21 | **0.007** | 2 | 4.70 | 0.095 | 1 | 0.27 | 0.602 | 2 | 22.87 | **0.000** | 2 | 15.21 | **0.000** |
| phen2 | GLMM | - | 1 | 10.68 | **0.001** | 1 | 19.81 | **0.000** | 2 | 7.04 | **0.030** | 1 | 116.70 | **0.000** | 2 | 45.00 | **0.000** | 2 | 9.12 | **0.010** |
| biomass1 | LMM | log | 1 | 2.96 | 0.085 | 1 | 0.45 | 0.504 | - | - | - | 1 | 5.74 | **0.017** | - | - | - | - | - | - |
| biomass2 | LMM | log | - | - | - | 1 | 11.10 | **0.001** | - | - | - | - | - | - | - | - | - | - | - | - |
| totbiomass | LMM | log | - | - | - | 1 | 6.18 | **0.013** | - | - | - | - | - | - | - | - | - | - | - | - |
| capitula1 | LMM | sqrt | - | - | - | - | - | - | - | - | - | - | - | - | - | - | - | - | - | - |
| capitula2 | LMM | sqrt | - | - | - | - | - | - | - | - | - | - | - | - | - | - | - | - | - | - |
| totcapitula | LMM | sqrt | - | - | - | - | - | - | - | - | - | - | - | - | - | - | - | - | - | - |
| flowperhead1 | LMM | - | - | - | - | - | - | - | - | - | - | - | - | - | - | - | - | - | - | - |
| flowperhead2 | LMM | - | 1 | 16.39 | **0.000** | - | - | - | - | - | - | - | - | - | - | - | - | - | - | - |
| flowers1 | LMM | log | 1 | 1.78 | 0.182 | 1 | 1.05 | 0.306 | - | - | - | - | - | - | 2 | 6.13 | **0.013** | - | - | - |
| flowers2 | LMM | log | - | - | - | - | - | - | - | - | - | - | - | - | - | - | - | - | - | - |
| totflowers | LMM | log | - | - | - | - | - | - | - | - | - | - | - | - | - | - | - | - | - | - |

**Legend**: C: carbon content; N: nitrogen content; SLA : specific leaf area; LDMC: leaf dry matter content; SC: stomatal conductance; ΔC: carbon isotope discrimination; surv: survival; presros: presence of accessory rosettes; ros: number of accessory rosettes; bolt: bolting; shoots: number of shoots; height: length of longest shoot; phen: phenology index; biomass: biomass per year; totbiomass: cumulative biomass; capitula: number of capitula per year; totcapitula: cumulative number of capitula; flowperhead: number of fertile flowers per flowerhead; flowers: number of flowers per year; totflowers: cumulative number of flowers; suffix “1”: 2009; suffix “2”: 2010; trans.: data transformation; df: difference in degrees of freedom between the two models (LRT); Chisq: Chi-squared; *P*: *P*-value.
