# Supplementary material for: Increased Phenotypic Plasticity to Climate May Have Boosted the Invasion Success of Polyploid Centaurea stoebe
Source: PLoS One. 2012 Nov 20;7(11):e50284. doi: 10.1371/journal.pone.0050284 (PMC3502303; doi:10.1371/journal.pone.0050284)
Supplement: Table S4 — Adaptive significance of phenotypic plasticity. Summary of tests on adaptive significance of phenotypic plasticity of C. stoebe in traits in response to site conditions (significant results highlighted in bold) based on different fitness measures (biomass, number of capitula and flowers; cumulative and for individual years). (DOC) [file pone.0050284.s005.doc]

**Supporting Table S4**

|  |  | **cumulative fitness** | | | | | | | | | | | | **fitness in individual years** | | | | | | | | | | | |  |
| --- | --- | --- | --- | --- | --- | --- | --- | --- | --- | --- | --- | --- | --- | --- | --- | --- | --- | --- | --- | --- | --- | --- | --- | --- | --- | --- |
|  |  | **totbiomass** | | | | **totcapitula** | | | | **totflowers** | | | | **biomass** | | | | **capitula** | | | | **flowers** | | | |  |
| **trait** | **treat.** | **est.** | **s.e.** | ***P*** | **df** | **est.** | **s.e.** | ***P*** | **df** | **est.** | **s.e.** | ***P*** | **df** | **est.** | **s.e.** | ***P*** | **df** | **est.** | **s.e.** | ***P*** | **df** | **est.** | **s.e.** | ***P*** | **df** | **adapt.sig.** |
| C | s | -0.20 | 0.52 | 0.704 | 22 | 1.31 | 0.46 | **0.010** | 22 | -0.20 | 0.53 | 0.708 | 22 | -0.57 | 0.49 | 0.261 | 22 | 1.06 | 0.49 | **0.041** | 22 | 0.02 | 0.52 | 0.962 | 22 | **+ +** |
| N | s | -0.11 | 0.48 | 0.822 | 22 | 1.19 | 0.42 | **0.010** | 22 | -0.34 | 0.49 | 0.491 | 22 | -0.49 | 0.46 | 0.299 | 22 | 0.95 | 0.45 | **0.046** | 22 | -0.21 | 0.49 | 0.669 | 22 | **+ +** |
| SLA1 | s | 0.30 | 0.55 | 0.593 | 19 | 1.57 | 0.47 | **0.003** | 19 | 0.02 | 0.57 | 0.969 | 19 | 0.28 | 0.48 | 0.576 | 8 | -0.27 | 0.47 | 0.578 | 8 | 0.11 | 0.58 | 0.853 | 6 | **+** |
| SLA2 | s | -0.01 | 0.50 | 0.989 | 22 | 1.42 | 0.42 | **0.003** | 22 | -0.10 | 0.51 | 0.852 | 22 | -0.43 | 0.49 | 0.387 | 22 | 1.16 | 0.45 | **0.018** | 22 | -0.04 | 0.52 | 0.934 | 22 | **+ +** |
| LDMC1 | s | 0.13 | 0.54 | 0.818 | 19 | 1.43 | 0.47 | **0.007** | 19 | 0.04 | 0.53 | 0.939 | 19 | 0.57 | 0.32 | 0.110 | 8 | 0.18 | 0.35 | 0.621 | 8 | 0.52 | 0.32 | 0.155 | 6 | **+** |
| LDMC2 | s | -0.06 | 0.46 | 0.904 | 22 | 1.50 | 0.44 | **0.002** | 22 | -0.09 | 0.53 | 0.867 | 22 | -0.49 | 0.47 | 0.308 | 22 | 1.22 | 0.48 | **0.018** | 22 | -0.08 | 0.54 | 0.880 | 22 | **+ +** |
| SC1 | s | -0.11 | 0.49 | 0.822 | 22 | 1.18 | 0.46 | **0.017** | 22 | -0.29 | 0.51 | 0.574 | 22 | -0.54 | 0.70 | 0.459 | 10 | -0.34 | 0.75 | 0.663 | 10 | -1.54 | 0.75 | 0.073 | 8 | **+** |
| SC2 | s | 0.34 | 0.65 | 0.606 | 15 | 0.99 | 0.59 | 0.113 | 15 | 0.04 | 0.66 | 0.953 | 15 | 0.18 | 0.63 | 0.782 | 15 | 0.91 | 0.59 | 0.142 | 15 | 0.23 | 0.60 | 0.711 | 15 |  |
| ΔC | s | 0.15 | 0.60 | 0.809 | 22 | 1.61 | 0.53 | **0.006** | 22 | 0.21 | 0.58 | 0.726 | 22 | -0.26 | 0.58 | 0.662 | 22 | 1.37 | 0.56 | **0.022** | 22 | 0.30 | 0.59 | 0.616 | 22 | **+ +** |
| ros1 | s | 0.19 | 0.25 | 0.446 | 76 | 0.76 | 0.21 | **0.001** | 76 | -0.76 | 0.30 | **0.013** | 62 | -0.62 | 0.48 | 0.200 | 47 | -0.06 | 0.48 | 0.896 | 47 | -0.95 | 0.46 | **0.046** | 43 | **+ / - -** |
| ros2 | s | -1.73 | 1.67 | 0.377 | 3 | -1.98 | 1.59 | 0.301 | 3 | -2.13 | 2.13 | 0.423 | 2 | -1.72 | 1.43 | 0.316 | 3 | -2.06 | 1.24 | 0.195 | 3 | - | - | - | - |  |
| shoots1 | s | 0.61 | 0.53 | 0.262 | 26 | 1.37 | 0.49 | **0.009** | 26 | -0.12 | 0.73 | 0.876 | 24 | -0.10 | 0.57 | 0.865 | 26 | 0.70 | 0.54 | 0.210 | 26 | -0.58 | 0.67 | 0.395 | 24 | **+** |
| shoots2 | s | -0.74 | 0.29 | **0.013** | 76 | -0.03 | 0.27 | 0.913 | 76 | -1.40 | 0.36 | **0.000** | 61 | -0.94 | 0.32 | **0.004** | 76 | -0.25 | 0.28 | 0.367 | 76 | -0.50 | 0.49 | 0.311 | 38 | **- - -** |
| height1 | s | 0.45 | 0.44 | 0.325 | 26 | 1.27 | 0.51 | **0.020** | 26 | -0.34 | 0.68 | 0.626 | 24 | -0.52 | 0.48 | 0.289 | 26 | 0.33 | 0.57 | 0.565 | 26 | -0.81 | 0.59 | 0.183 | 24 | **+** |
| height2 | s | 0.50 | 0.22 | **0.026** | 75 | 0.98 | 0.23 | **0.000** | 75 | -0.63 | 0.32 | 0.054 | 59 | 0.22 | 0.23 | 0.329 | 75 | 0.83 | 0.23 | **0.001** | 75 | 0.39 | 0.44 | 0.376 | 37 | **+ + +** |
| phen1 | s | 1.00 | 0.44 | **0.033** | 23 | 1.51 | 0.59 | **0.018** | 23 | -0.23 | 0.68 | 0.734 | 23 | -0.33 | 0.52 | 0.533 | 23 | -0.05 | 0.69 | 0.938 | 23 | -0.52 | 0.56 | 0.363 | 23 | **+ +** |
| phen2 | s | 0.50 | 0.26 | 0.057 | 75 | 1.05 | 0.25 | **0.000** | 75 | -0.55 | 0.34 | 0.112 | 58 | 0.22 | 0.27 | 0.419 | 75 | 0.88 | 0.27 | **0.001** | 75 | 0.41 | 0.44 | 0.362 | 37 | **+ +** |
| C | w | -0.41 | 0.51 | 0.435 | 22 | 1.09 | 0.46 | **0.027** | 22 | 0.10 | 0.51 | 0.850 | 22 | -0.73 | 0.50 | 0.161 | 22 | 0.88 | 0.49 | 0.086 | 22 | 0.16 | 0.52 | 0.753 | 22 | **+** |
| N | w | -0.48 | 0.49 | 0.335 | 22 | 0.98 | 0.46 | **0.044** | 22 | 0.01 | 0.51 | 0.984 | 22 | -0.77 | 0.48 | 0.120 | 22 | 0.79 | 0.48 | 0.115 | 22 | 0.04 | 0.51 | 0.939 | 22 | **+** |
| SLA1 | w | -0.24 | 0.44 | 0.589 | 24 | 1.01 | 0.43 | **0.029** | 24 | 0.31 | 0.48 | 0.517 | 24 | 0.40 | 1.40 | 0.785 | 7 | 1.11 | 1.44 | 0.467 | 7 | 0.86 | 0.33 | **0.048** | 5 | **+ +** |
| SLA2 | w | -0.56 | 0.45 | 0.228 | 24 | 0.89 | 0.43 | **0.047** | 24 | 0.05 | 0.46 | 0.914 | 24 | -0.84 | 0.44 | 0.067 | 24 | 0.69 | 0.45 | 0.135 | 24 | 0.04 | 0.47 | 0.938 | 24 | **+** |
| LDMC1 | w | -0.25 | 0.47 | 0.604 | 24 | 0.91 | 0.45 | 0.057 | 24 | 0.22 | 0.48 | 0.652 | 24 | -0.56 | 1.09 | 0.625 | 7 | 0.37 | 1.08 | 0.743 | 7 | 1.07 | 1.11 | 0.380 | 5 |  |
| LDMC2 | w | -0.63 | 0.46 | 0.185 | 24 | 1.00 | 0.44 | **0.033** | 24 | 0.06 | 0.48 | 0.905 | 24 | -0.94 | 0.44 | **0.042** | 24 | 0.77 | 0.46 | 0.111 | 24 | -0.01 | 0.49 | 0.983 | 24 | **+ / -** |
| SC1 | w | -0.69 | 0.43 | 0.120 | 24 | 0.94 | 0.45 | **0.046** | 24 | -0.02 | 0.47 | 0.965 | 24 | -0.40 | 1.75 | 0.827 | 9 | 2.39 | 1.57 | 0.164 | 9 | -1.42 | 0.72 | 0.089 | 7 | **+** |
| SC2 | w | 0.33 | 0.55 | 0.559 | 13 | 0.78 | 0.51 | 0.152 | 13 | 0.05 | 0.59 | 0.940 | 13 | 0.06 | 0.41 | 0.888 | 13 | 0.68 | 0.49 | 0.189 | 13 | -0.11 | 0.54 | 0.840 | 13 |  |
| ΔC | w | 0.08 | 0.52 | 0.876 | 22 | 1.67 | 0.44 | **0.001** | 22 | 0.66 | 0.49 | 0.196 | 22 | -0.26 | 0.50 | 0.606 | 22 | 1.47 | 0.47 | **0.005** | 22 | 0.60 | 0.51 | 0.251 | 22 | **+ +** |
| ros1 | w | 0.19 | 0.26 | 0.464 | 76 | 0.75 | 0.22 | **0.001** | 76 | -0.58 | 0.30 | 0.061 | 54 | -0.34 | 0.46 | 0.471 | 43 | 0.20 | 0.47 | 0.677 | 43 | -1.18 | 0.57 | **0.046** | 36 | **+ / -** |
| ros2 | w | 0.13 | 0.38 | 0.754 | 4 | 0.03 | 0.44 | 0.947 | 4 | -0.13 | 0.49 | 0.810 | 3 | 0.13 | 0.38 | 0.739 | 4 | 0.05 | 0.44 | 0.908 | 4 | - | - | - | - |  |
| shoots1 | w | 0.11 | 0.71 | 0.876 | 22 | 0.92 | 0.65 | 0.173 | 22 | -0.94 | 0.61 | 0.138 | 21 | -1.54 | 0.59 | **0.017** | 22 | -0.86 | 0.56 | 0.140 | 22 | -1.84 | 0.48 | **0.001** | 21 | **- -** |
| shoots2 | w | -0.66 | 0.28 | **0.024** | 75 | 0.25 | 0.29 | 0.386 | 75 | -1.22 | 0.37 | **0.002** | 51 | -0.79 | 0.31 | **0.012** | 75 | 0.10 | 0.30 | 0.728 | 75 | -0.48 | 0.45 | 0.300 | 31 | **- - -** |
| height1 | w | 0.02 | 0.66 | 0.975 | 22 | 1.05 | 0.63 | 0.110 | 22 | -1.08 | 0.65 | 0.112 | 21 | -1.75 | 0.43 | **0.001** | 22 | -1.61 | 0.59 | **0.012** | 22 | -2.18 | 0.45 | **0.000** | 21 | **- - -** |
| height2 | w | 0.50 | 0.21 | **0.020** | 75 | 0.95 | 0.24 | **0.000** | 75 | -0.52 | 0.35 | 0.145 | 51 | 0.23 | 0.21 | 0.292 | 75 | 0.80 | 0.24 | **0.001** | 75 | 0.59 | 0.41 | 0.162 | 31 | **+ + +** |
| phen1 | w | -0.39 | 0.71 | 0.594 | 19 | 0.57 | 0.61 | 0.363 | 19 | -1.24 | 0.64 | 0.068 | 19 | -1.78 | 0.57 | **0.006** | 19 | -1.72 | 0.66 | **0.017** | 19 | -2.24 | 0.54 | **0.001** | 19 | **- - -** |
| phen2 | w | 0.27 | 0.28 | 0.339 | 72 | 0.86 | 0.25 | **0.001** | 72 | -0.44 | 0.38 | 0.256 | 48 | -0.01 | 0.28 | 0.985 | 72 | 0.70 | 0.26 | **0.009** | 72 | 0.33 | 0.44 | 0.457 | 31 | **+ +** |
| C | n | -0.13 | 0.52 | 0.801 | 22 | 0.98 | 0.49 | 0.057 | 22 | -0.46 | 0.52 | 0.385 | 22 | -0.46 | 0.51 | 0.373 | 22 | 0.79 | 0.50 | 0.132 | 22 | -0.12 | 0.53 | 0.823 | 22 |  |
| N | n | 0.00 | 0.54 | 0.993 | 22 | 1.17 | 0.49 | **0.025** | 22 | -0.26 | 0.53 | 0.634 | 22 | -0.30 | 0.52 | 0.578 | 22 | 1.01 | 0.50 | 0.057 | 22 | 0.00 | 0.54 | 1.000 | 22 | **+** |
| SLA1 | n | 0.01 | 0.49 | 0.983 | 22 | 0.91 | 0.51 | 0.088 | 22 | 0.08 | 0.53 | 0.877 | 22 | 0.27 | 0.32 | 0.425 | 9 | 0.29 | 0.33 | 0.408 | 9 | 0.35 | 0.34 | 0.345 | 7 |  |
| SLA2 | n | -0.12 | 0.52 | 0.824 | 22 | 1.04 | 0.49 | **0.044** | 22 | -0.31 | 0.52 | 0.556 | 22 | -0.46 | 0.51 | 0.380 | 22 | 0.86 | 0.50 | 0.103 | 22 | -0.18 | 0.52 | 0.726 | 22 | **+** |
| LDMC1 | n | -0.25 | 0.54 | 0.649 | 22 | 0.61 | 0.53 | 0.261 | 22 | 0.00 | 0.56 | 0.995 | 22 | -0.19 | 0.34 | 0.587 | 9 | -0.25 | 0.36 | 0.518 | 9 | -0.29 | 0.31 | 0.374 | 7 |  |
| LDMC2 | n | -0.52 | 0.57 | 0.372 | 22 | 0.86 | 0.56 | 0.136 | 22 | -0.43 | 0.60 | 0.486 | 22 | -0.82 | 0.55 | 0.148 | 22 | 0.68 | 0.57 | 0.250 | 22 | -0.37 | 0.59 | 0.537 | 22 |  |
| SC1 | n | -0.47 | 0.49 | 0.349 | 22 | 0.64 | 0.51 | 0.217 | 22 | -0.72 | 0.49 | 0.153 | 22 | 0.11 | 0.30 | 0.706 | 11 | 0.21 | 0.29 | 0.489 | 11 | 0.24 | 0.31 | 0.455 | 9 |  |
| SC2 | n | 0.01 | 0.47 | 0.990 | 18 | 0.78 | 0.50 | 0.137 | 18 | -0.38 | 0.50 | 0.452 | 17 | -0.17 | 0.45 | 0.712 | 18 | 0.67 | 0.50 | 0.197 | 18 | -0.28 | 0.49 | 0.577 | 17 |  |
| ΔC | n | -0.21 | 0.60 | 0.728 | 22 | 0.73 | 0.56 | 0.206 | 22 | -0.70 | 0.58 | 0.242 | 22 | -0.45 | 0.59 | 0.450 | 22 | 0.58 | 0.58 | 0.324 | 22 | -0.36 | 0.59 | 0.551 | 22 |  |
| ros1 | n | 0.25 | 0.26 | 0.342 | 76 | 0.76 | 0.25 | **0.003** | 76 | -0.68 | 0.33 | **0.043** | 55 | -0.70 | 0.53 | 0.194 | 44 | -0.29 | 0.56 | 0.600 | 45 | -0.85 | 0.54 | 0.128 | 38 | **+ / -** |
| ros2 | n | - | - | - | - | - | - | - | - | - | - | - | - | - | - | - | - | - | - | - | - | - | - | - | - |  |
| shoots1 | n | -0.02 | 0.58 | 0.979 | 22 | 0.69 | 0.56 | 0.230 | 22 | -1.22 | 0.66 | 0.079 | 21 | -2.51 | 0.58 | **0.000** | 22 | -2.31 | 0.52 | **0.000** | 22 | -2.77 | 0.50 | **0.000** | 21 | **- - -** |
| shoots2 | n | -0.57 | 0.26 | **0.032** | 76 | -0.08 | 0.25 | 0.764 | 76 | -1.08 | 0.39 | **0.008** | 55 | -0.69 | 0.30 | **0.024** | 76 | -0.23 | 0.26 | 0.382 | 76 | -0.51 | 0.48 | 0.295 | 35 | **- - -** |
| height1 | n | -0.02 | 0.58 | 0.967 | 22 | 0.41 | 0.62 | 0.519 | 22 | -1.16 | 0.68 | 0.105 | 21 | -2.37 | 0.45 | **0.000** | 22 | -2.61 | 0.59 | **0.000** | 22 | -2.72 | 0.48 | **0.000** | 21 | **- - -** |
| height2 | n | 0.47 | 0.23 | **0.047** | 75 | 0.93 | 0.25 | **0.000** | 75 | -0.41 | 0.35 | 0.250 | 54 | 0.20 | 0.23 | 0.394 | 75 | 0.79 | 0.26 | **0.003** | 75 | 0.35 | 0.44 | 0.421 | 35 | **+ + +** |
| phen1 | n | 0.00 | 0.72 | 0.995 | 20 | 0.37 | 0.69 | 0.600 | 20 | -1.18 | 0.74 | 0.126 | 19 | -2.13 | 0.47 | **0.000** | 20 | -2.41 | 0.59 | **0.001** | 20 | -2.49 | 0.50 | **0.000** | 19 | **- - -** |
| phen2 | n | 0.24 | 0.28 | 0.396 | 75 | 0.79 | 0.26 | **0.004** | 75 | -0.60 | 0.37 | 0.109 | 54 | -0.03 | 0.29 | 0.928 | 75 | 0.64 | 0.27 | **0.023** | 75 | 0.06 | 0.47 | 0.896 | 34 | **+ +** |

**Legend:** trait: (C: carbon content; N: nitrogen content; SLA : specific leaf area; LDMC: leaf dry matter content; SC: stomatal conductance; ΔC: carbon isotope discrimination; ros: number of accessory rosettes; shoots: number of shoots; height: length of longest shoot; phen: phenology index; suffix “1”: 2009; suffix “2”: 2010); treat.: treatment (s: site; w: water; n: nutrients); est.: parameter estimate (slope of absolute plasticity); s.e.: standard error; *P*: *P*-value; df: degrees of freedom; adapt.sig.: adaptive significance (“+”: adaptive;” –“: maladaptive).
