# Supplementary material for: Increased Phenotypic Plasticity to Climate May Have Boosted the Invasion Success of Polyploid Centaurea stoebe
Source: PLoS One. 2012 Nov 20;7(11):e50284. doi: 10.1371/journal.pone.0050284 (PMC3502303; doi:10.1371/journal.pone.0050284)
Supplement: Table S5 — Comparisons of relative phenotypic plasticity among geo-cytotypes. Differences in relative phenotypic plasticity indices (PI) between (a) European diploid and European tetraploid and (b) European tetraploid and North American tetraploid C. stoebe in response to different experimental treatments (site, water, nutrients). (DOC) [file pone.0050284.s006.doc]

**Supporting Table S5**

| **(a) EU 2x *vs*. EU 4x** | | |  |  |  |  |  |  |  |  |  |  |  |  |  |  |  |  |  |  |  |
| --- | --- | --- | --- | --- | --- | --- | --- | --- | --- | --- | --- | --- | --- | --- | --- | --- | --- | --- | --- | --- | --- |
|  | **site** | | | | | | | **water** | | | | | | | **nutrients** | | | | | | |
|  | **# seed fam.** | | **PI** |  | **PI** |  |  | **# seed fam.** | | **PI** |  | **PI** |  |  | **# seed fam.** | | **PI** |  | **PI** |  |  |
| **trait** | **2x** | **4x** | **2x** |  | **4x** | **LR** | ***P*** | **2x** | **4x** | **2x** |  | **4x** | **LR** | ***P*** | **2x** | **4x** | **2x** |  | **4x** | **LR** | ***P*** |
| C | 9 | 9 | 0.019 |  | 0.011 | 2.52 | 0.113 | 9 | 9 | 0.002 |  | 0.001 | 0.04 | 0.842 | 9 | 9 | 0.008 |  | 0.002 | 0.60 | 0.439 |
| N | 9 | 9 | 0.233 |  | 0.332 | 2.71 | 0.100 | 9 | 9 | 0.038 |  | 0.089 | 0.56 | 0.455 | 9 | 9 | 0.051 |  | 0.027 | 0.07 | 0.791 |
| SLA1 | 7 | 9 | 0.124 |  | 0.161 | 0.19 | 0.664 | 11 | 9 | 0.041 |  | -0.003 | 0.64 | 0.425 | 9 | 9 | 0.030 |  | -0.013 | 0.85 | 0.357 |
| SLA2 | 9 | 9 | 0.093 | < | 0.222 | 6.03 | **0.014** | 11 | 9 | 0.058 |  | 0.008 | 0.13 | 0.723 | 9 | 9 | 0.003 |  | 0.046 | 0.82 | 0.366 |
| LDMC1 | 7 | 9 | 0.124 |  | 0.130 | 0.01 | 0.913 | 11 | 9 | -0.009 |  | 0.016 | 0.21 | 0.644 | 9 | 9 | 0.000 |  | 0.071 | 1.12 | 0.290 |
| LDMC2 | 9 | 9 | 0.104 | < | 0.172 | 4.05 | **0.044** | 11 | 9 | 0.022 |  | 0.001 | 0.12 | 0.729 | 9 | 9 | 0.038 |  | 0.008 | 0.52 | 0.471 |
| SC1 | 9 | 9 | 0.932 |  | 0.677 | 2.11 | 0.147 | 11 | 9 | 0.320 |  | 0.147 | 0.22 | 0.641 | 9 | 9 | -0.123 |  | -0.067 | 0.03 | 0.859 |
| SC2 | 5 | 7 | 0.311 | < | 0.956 | 4.50 | **0.034** | 5 | 5 | -0.081 |  | 0.313 | 1.12 | 0.291 | 7 | 8 | -0.085 |  | 0.264 | 0.76 | 0.384 |
| ΔC | 9 | 9 | 0.025 |  | 0.008 | 0.81 | 0.368 | 9 | 9 | 0.001 |  | 0.017 | 1.17 | 0.279 | 9 | 9 | 0.001 |  | 0.016 | 0.98 | 0.323 |
| ros1 | 27 | 27 | 0.501 |  | 0.471 | 0.04 | 0.838 | 27 | 27 | -0.097 |  | 0.041 | 0.86 | 0.353 | 27 | 27 | 0.043 |  | -0.088 | 1.00 | 0.317 |
| ros2 | - | - | - |  | - | - | - | 27 | 27 | -0.097 |  | 0.041 | 0.86 | 0.353 | - | - | - |  | - | - | - |
| shoots1 | 4 | 11 | 0.003 |  | 0.501 | 1.45 | 0.228 | - | - | - |  | - | - | - | 2 | 12 | 0.744 |  | 0.183 | 1.15 | 0.284 |
| shoots2 | 27 | 27 | 0.430 |  | 0.598 | 1.92 | 0.166 | 26 | 27 | -0.086 |  | 0.110 | 1.66 | 0.197 | 27 | 27 | -0.034 |  | 0.158 | 1.61 | 0.205 |
| height1 | 4 | 11 | 0.499 | > | -0.059 | 6.81 | **0.009** | 3 | 10 | 0.511 |  | 0.166 | 0.88 | 0.348 | 2 | 12 | -0.240 |  | 0.169 | 1.51 | 0.219 |
| height2 | 26 | 27 | 0.249 |  | 0.208 | 1.14 | 0.285 | 26 | 27 | 0.017 |  | 0.009 | 0.01 | 0.906 | 26 | 27 | 0.004 |  | -0.017 | 0.09 | 0.764 |
| phen1 | 3 | 11 | -0.160 |  | 0.698 | 2.31 | 0.129 | 3 | 10 | -1.381 | < | 0.327 | 4.50 | **0.034** | 2 | 10 | 0.401 |  | -0.059 | 0.30 | 0.584 |
| phen2 | 26 | 27 | 0.125 | < | 0.719 | 22.28 | **0.000** | 26 | 25 | 0.004 |  | 0.052 | 0.20 | 0.659 | 26 | 27 | 0.074 |  | -0.145 | 3.07 | 0.080 |
| biomass1 | 3 | 11 | 0.972 |  | 0.374 | 1.80 | 0.180 | 3 | 10 | 0.936 |  | 0.056 | 2.84 | 0.092 | 2 | 12 | 0.788 |  | -0.107 | 1.32 | 0.251 |
| biomass2 | 27 | 27 | 0.801 |  | 0.865 | 0.29 | 0.589 | 26 | 27 | -0.030 |  | 0.045 | 0.16 | 0.688 | 27 | 27 | -0.036 |  | 0.028 | 0.10 | 0.751 |
| totbiomass | 27 | 27 | 0.886 |  | 0.774 | 1.16 | 0.282 | 27 | 27 | -0.071 |  | -0.004 | 0.14 | 0.705 | 27 | 27 | 0.014 |  | -0.074 | 0.28 | 0.594 |
| capitula1 | 4 | 11 | -0.080 |  | 0.756 | 2.69 | 0.101 | 3 | 10 | 1.340 | > | -0.080 | 5.34 | **0.021** | 2 | 12 | 0.680 |  | 0.013 | 0.54 | 0.463 |
| capitula2 | 26 | 27 | 0.448 |  | 0.303 | 0.71 | 0.400 | 26 | 26 | -0.017 |  | -0.006 | 0.00 | 0.961 | 26 | 27 | -0.068 |  | 0.091 | 0.52 | 0.472 |
| totcapitula | 27 | 27 | 0.528 |  | 0.260 | 2.76 | 0.097 | 27 | 27 | -0.078 |  | -0.059 | 0.01 | 0.929 | 27 | 27 | -0.002 |  | 0.041 | 0.05 | 0.820 |
| flowperhead1 | 3 | 10 | -0.002 |  | 0.076 | 0.24 | 0.621 | 1 | 9 | -0.052 |  | 0.024 | 0.16 | 0.692 | 2 | 8 | -0.164 |  | 0.088 | 1.63 | 0.202 |
| flowperhead2 | 9 | 7 | -0.013 |  | 0.058 | 1.18 | 0.278 | 10 | 9 | 0.094 |  | 0.055 | 0.23 | 0.629 | 9 | 9 | 0.066 |  | 0.044 | 0.08 | 0.773 |
| flowers1 | 3 | 10 | 0.901 |  | 0.621 | 0.37 | 0.542 | 1 | 9 | 0.443 |  | 0.265 | 0.05 | 0.827 | 2 | 8 | 0.542 |  | 0.071 | 0.45 | 0.504 |
| flowers2 | 9 | 7 | 0.515 |  | 0.154 | 0.78 | 0.379 | 10 | 9 | -0.103 |  | -0.131 | 0.00 | 0.958 | 9 | 9 | 0.332 |  | 0.202 | 0.08 | 0.771 |
| totflowers | 12 | 19 | 0.616 |  | 0.324 | 0.71 | 0.400 | 11 | 14 | -0.053 |  | 0.071 | 0.08 | 0.777 | 11 | 14 | 0.181 |  | 0.123 | 0.03 | 0.871 |
| **(b) EU 4x *vs*. NA 4x** | | |  |  |  |  |  |  |  |  |  |  |  |  |  |  |  |  |  |  |  |
|  | **site** | | | | | | | **water** | | | | | | | **nutrients** | | | | | | |
|  | **# seed fam.** | | **PI** |  | **PI** |  |  | **# seed fam.** | | **PI** |  | **PI** |  |  | **# seed fam.** | | **PI** |  | **PI** |  |  |
| **trait** | **EU** | **NA** | **EU** |  | **NA** | **LR** | ***P*** | **EU** | **NA** | **EU** |  | **NA** | **LR** | ***P*** | **EU** | **NA** | **EU** |  | **NA** | **LR** | ***P*** |
| C | 9 | 9 | 0.011 |  | 0.026 | 3.64 | 0.057 | 9 | 9 | -0.001 |  | 0.003 | 0.13 | 0.717 | 9 | 9 | -0.002 |  | 0.002 | 0.22 | 0.636 |
| N | 9 | 9 | 0.332 |  | 0.296 | 0.39 | 0.534 | 9 | 9 | 0.089 |  | -0.004 | 1.51 | 0.220 | 9 | 9 | 0.027 |  | -0.032 | 0.50 | 0.479 |
| SLA1 | 9 | 8 | 0.161 |  | 0.219 | 0.27 | 0.605 | 9 | 9 | -0.003 |  | 0.035 | 0.31 | 0.578 | 9 | 9 | 0.013 |  | -0.010 | 0.20 | 0.651 |
| SLA2 | 9 | 9 | 0.222 |  | 0.174 | 0.95 | 0.331 | 9 | 9 | 0.008 |  | 0.028 | 0.16 | 0.685 | 9 | 9 | 0.046 |  | -0.013 | 1.24 | 0.266 |
| LDMC1 | 9 | 8 | 0.130 |  | 0.150 | 0.20 | 0.657 | 9 | 9 | 0.016 |  | 0.028 | 0.04 | 0.835 | 9 | 9 | 0.071 | > | -0.025 | 3.93 | **0.047** |
| LDMC2 | 9 | 9 | 0.172 |  | 0.134 | 1.39 | 0.239 | 9 | 9 | 0.001 |  | 0.058 | 3.27 | 0.071 | 9 | 9 | 0.008 |  | 0.017 | 0.06 | 0.806 |
| SC1 | 9 | 9 | 0.677 |  | 0.541 | 0.45 | 0.501 | 9 | 9 | 0.147 |  | 0.216 | 0.09 | 0.759 | 9 | 9 | -0.067 |  | -0.037 | 0.01 | 0.907 |
| SC2 | 7 | 8 | 0.956 |  | 0.938 | 0.00 | 0.950 | 5 | 8 | 0.313 |  | -0.108 | 1.33 | 0.249 | 8 | 8 | 0.264 |  | -0.089 | 0.78 | 0.377 |
| ΔC | 9 | 9 | 0.008 |  | 0.040 | 2.79 | 0.095 | 9 | 9 | 0.017 |  | -0.006 | 3.00 | 0.083 | 9 | 9 | -0.016 | < | 0.016 | 4.13 | **0.042** |
| ros1 | 27 | 27 | 0.471 |  | 0.476 | 0.00 | 0.966 | 27 | 27 | -0.041 |  | 0.130 | 1.41 | 0.235 | 27 | 27 | -0.088 |  | -0.021 | 0.21 | 0.645 |
| ros2 | 1 | 6 | 0.074 |  | 0.609 | 0.75 | 0.386 | 1 | 7 | 0.000 |  | 0.204 | 0.05 | 0.826 | - | - | - |  | - | - | - |
| shoots1 | 11 | 16 | 0.501 |  | 0.205 | 1.44 | 0.231 | 10 | 14 | 0.106 |  | -0.001 | 0.16 | 0.689 | 12 | 13 | 0.183 |  | 0.149 | 0.01 | 0.907 |
| shoots2 | 27 | 27 | 0.598 |  | 0.595 | 0.00 | 0.973 | 27 | 27 | 0.110 |  | 0.039 | 0.24 | 0.626 | 27 | 27 | 0.158 |  | -0.021 | 2.31 | 0.128 |
| height1 | 11 | 16 | 0.059 |  | 0.115 | 0.17 | 0.684 | 10 | 14 | 0.166 |  | -0.133 | 1.43 | 0.232 | 12 | 13 | 0.169 |  | -0.049 | 1.76 | 0.184 |
| height2 | 27 | 27 | 0.208 |  | 0.229 | 0.27 | 0.602 | 27 | 27 | -0.009 |  | -0.012 | 0.00 | 0.958 | 27 | 27 | 0.017 |  | 0.004 | 0.03 | 0.858 |
| phen1 | 11 | 14 | 0.698 |  | 0.524 | 0.22 | 0.637 | 10 | 11 | 0.327 |  | 0.109 | 0.19 | 0.664 | 10 | 13 | -0.059 |  | 0.082 | 0.13 | 0.715 |
| phen2 | 27 | 27 | 0.719 | > | 0.159 | 21.26 | **0.000** | 25 | 26 | 0.052 |  | -0.023 | 0.47 | 0.491 | 27 | 27 | 0.145 |  | 0.027 | 0.95 | 0.330 |
| biomass1 | 11 | 15 | 0.374 |  | 0.165 | 0.38 | 0.539 | 10 | 12 | 0.056 |  | 0.162 | 0.07 | 0.792 | 12 | 13 | 0.107 |  | 0.037 | 0.03 | 0.873 |
| biomass2 | 27 | 27 | 0.865 |  | 0.861 | 0.00 | 0.968 | 27 | 27 | 0.045 |  | -0.003 | 0.07 | 0.791 | 27 | 27 | 0.028 |  | -0.120 | 0.59 | 0.442 |
| totbiomass | 27 | 27 | 0.774 |  | 0.714 | 0.36 | 0.547 | 27 | 27 | 0.004 |  | 0.123 | 0.54 | 0.461 | 27 | 27 | -0.074 |  | -0.042 | 0.05 | 0.829 |
| capitula1 | 11 | 16 | 0.756 |  | 0.590 | 0.24 | 0.626 | 10 | 14 | 0.080 |  | 0.319 | 0.27 | 0.603 | 12 | 13 | -0.013 |  | 0.129 | 0.10 | 0.755 |
| capitula2 | 27 | 27 | 0.303 |  | 0.330 | 0.03 | 0.874 | 26 | 26 | -0.006 |  | 0.001 | 0.00 | 0.974 | 27 | 27 | 0.091 |  | -0.102 | 0.74 | 0.390 |
| totcapitula | 27 | 27 | 0.260 |  | 0.306 | 0.09 | 0.761 | 27 | 27 | -0.059 |  | -0.056 | 0.00 | 0.985 | 27 | 27 | 0.041 |  | 0.006 | 0.03 | 0.853 |
| flowperhead1 | 10 | 12 | 0.076 |  | 0.034 | 0.13 | 0.718 | 9 | 9 | 0.024 |  | 0.035 | 0.01 | 0.919 | 8 | 13 | 0.088 |  | 0.001 | 0.48 | 0.488 |
| flowperhead2 | 7 | 6 | 0.050 |  | -0.129 | 0.68 | 0.410 | 9 | 9 | 0.055 |  | -0.057 | 1.63 | 0.201 | 9 | 8 | 0.044 |  | 0.025 | 0.08 | 0.782 |
| flowers1 | 10 | 12 | 0.621 |  | 0.218 | 1.11 | 0.293 | 9 | 9 | 0.265 |  | 0.095 | 0.14 | 0.710 | 8 | 12 | -0.071 |  | -0.057 | 0.00 | 0.976 |
| flowers2 | 7 | 6 | 0.154 |  | 0.067 | 0.03 | 0.853 | 9 | 9 | -0.131 |  | -0.222 | 0.03 | 0.873 | 9 | 8 | -0.202 |  | -0.383 | 0.14 | 0.710 |
| totflowers | 19 | 17 | 0.324 |  | -0.009 | 1.11 | 0.291 | 14 | 16 | 0.071 |  | 0.373 | 0.66 | 0.417 | 14 | 17 | 0.123 |  | -0.203 | 1.01 | 0.316 |

**Legend**: C: carbon content; N: nitrogen content; SLA : specific leaf area; LDMC: leaf dry matter content; SC: stomatal conductance; ΔC: carbon isotope discrimination; ros: number of accessory rosettes; shoots: number of shoots; height: length of longest shoot; phen: phenology index; biomass: biomass per year; totbiomass: cumulative biomass; capitula: number of capitula per year; totcapitula: cumulative number of capitula; flowperhead: number of fertile flowers per flowerhead; flowers: number of flowers per year; totflowers: cumulative number of flowers; suffix “1”: 2009; suffix “2”: 2010.
